# Application of recycled battery graphite decorated with poly hippuric acid/ multiwalled carbon nanotubes as an ecofriendly sensor for serotonin

**DOI:** 10.1038/s41598-024-80673-y

**Published:** 2024-11-26

**Authors:** Aya G. Abd El-Nasser, Mahmoud G. Metwally, Azza A. Shoukry, Rasha M. El Nashar

**Affiliations:** Chemistry Department, Faculty of Science, Cairo University, Giza, 12613 Egypt

**Keywords:** Serotonin, Sensors, Electropolymerization, Hippuric acid, Modified electrodes., Biomaterials, Electrocatalysis, Analytical chemistry, Electrochemistry, Green chemistry, Materials chemistry, Biomarkers

## Abstract

A novel modified sensor based on electropolymerization of hippuric acid (HA) using cyclic voltammetry within the potential window − 1 to 1.5 V for 10 cycles at a scan rate 100 mV s^−1^ over multiwalled carbon nanotubes (MWCNTs) on battery graphite electrode (BGE). Poly (HA)/MWCNTs/BGE sensor exhibited two linearity ranges 3.00 × 10^−3^ to 1.00 µM (5.29 × 10^−4^ – 0.18 µg/ml) and 5.00 to 1.00 × 10^3^ µM (0.88− 176.22 µg/ml) with limit of detection (LOD) of 0.06 × 10^−2^ µM (1.06 × 10^−4^ µg/ml) and limit of quantification (LOQ) of 2.00 × 10^−3^ µM (3.52 × 10^−4^ µg/ml). The poly (HA)/MWCNTs/BGE sensor was successfully applied to the determination of SER in the presence of tryptophan and in human blood serum with recovery ranges 98.31–105.47% with RSD values 3.02– 4.77%. Green chemistry metrics : national environmental index (NEMI), analytical greenness metric (AGREE), Raynie and Driver, green analytical procedure index (GAPI), and the analytical eco-scale were employed and indicated that the proposed sensor can be classified as an excellent green method, achieving an analytical eco-scale score of 84.

Serotonin (SER), also known as 5-hydroxytryptamine (5-HT), is an important catecholamine neurotransmitter in biological systems. It is produced by serotonergic neurons in the central nervous system (CNS) and enterochromaffin cells in the gastrointestinal tract. SER is essential in regulating various functions in the body, including mood, sleep, vomiting, cardiovascular activity and appetite^1–4^. The recognition of SER plays a crucial role in the diagnosis of specific conditions such as Down’s syndrome, Alzheimer’s and Huntington’s disease^2,5^.

Low SER levels results in a variety of symptoms of which, decreased appetite, reduced energy levels, mood swings, loss of interest^26^, Parkinson’s disease, and Alzheimer’s disease^7^. On the other hand, elevated levels of SER can potentially show fatal consequences and results in toxicity, known as serotonin syndrome^4,6^.

Several analytical methods have been reported in literature to quantify SER including spectrophotometry^8–11^, capillary electrophoresis^12^, high-performance liquid chromatography (HPLC)^13^, liquid chromatography (LC) combined with mass spectrometry^14,15^Ultrahigh-performance Liquid Chromatography Coupled with Tandem Mass Spectrometry (UHPLC–MS/MS)s^16,17,18^, UHPLC with fluorescence detection (FD)^19^, high-precision liquid chromatography with an electrochemical detector (HPLC-ECD)^20^, gas chromatography coupled with Mass Spectrometry (GC-MS)^21^. Nevertheless, most of these techniques require sophisticated expensive instruments, very long detection times and require expert and well qualified personal to conduct the procedures^1,2^, and this creates a demand for a more simple approach that can be efficiently used within min facilities and experience as part of possible application as point of care for patients.

During the past few decades, electrochemical techniques have garnered significant attention among various analytical methods due to their rapid response, high sensitivity, cost-effectiveness, accuracy, and ease of operation^22–24^.Various electrochemical sensors have been reported for the analysis of SER as an electroactive material employing cyclic voltammetry (CV) and differential pulse voltammetry (DPV) technique performed on carbon paste electrode (CPE)^6,25–28^, glassy carbon electrode (GCE)^2,29–31^, screen printed electrode (SPCE)^1,32^and graphite pencil electrode^33–36^ as the working electrodes.

To the best of our knowledge, there have been no published studies on the determination of SER involving graphite electrode made from waste batteries as the working electrode. Accordingly, one of the main goals of the current work is to investigate its applicability being inexpensive, and non-toxic, and its favourable electrochemical characteristics, including excellent electrical conductivity, ease of preparation, low toxicity, and the large surface area of the graphite rod^37^.

To resolve the problem of low sensitivity and selectivity on using a bare graphite electrode for a target analyte, conductive and/or selective materials are usually incorporated to increase the sensitivity and selectivity^38^. Based on literature, a variety of materials have been used to modify electrode’s surface for the purpose of detecting SER, recent examples of which: conducting polymers, amino acids as polyalanine^27^, polyglutamic acid^29^and poly(L-lysine)^33^, and electropolymerized dyes as poly-alizarin^39^, polymethylene blue^32^, poly (Bromocresol purple)^34^. Carbon nanomaterials like carbon dots (CDs)^21,33,40^, multi-walled carbon nanotubes (MWCNTs)^36,39,41^, reduced graphene oxide (rGO)^4,28,42^metals and metal oxide nanoparticles (NPs) like gold (AuNPs)^23,33^, silver (Ag NPs)^29^, palladium (Pd NPs)^40^, Nickel (Ni NPs)^31^, Fe_3_O_4_^2^, ZrO_2_^6^, TiO_2_NPs/AuNPs^1^ were also reported.

The utilization of carbon nanomaterials to modify bare electrodes was found to offer numerous advantages due to their significant surface-to-volume ratio, specific surface area, superior electrocatalytic activity, biocompatibility, enhanced adsorption properties and rapid electron transfer kinetics^38^. Both single-walled carbon nanotubes (SWCNTs) and multi-walled carbon nanotubes (MWCNTs) are widely employed and can be combined with other conducting materials to synergistically enhance the response^43–45^.

Conducting polymers are one of the most used modifiers in the fabrication of modified electrodes and have functional groups that can be oxidized or reduced^38^, owing to their excellent conductivity, chemical durability, mechanical stability, and ease of preparation^46^. Additionally, they have high sensitivity, more active sites, homogeneity, stability of the polymer films, and strong adhesion to the electrode surface^5,47^. Among the recently applied conducting polymers, amino acids are able to form conductive polymer films with high electrocatalytic activity on the surface of bare electrode through electropolymerization^48^, and the electropolymerization can provide numerous advantages, including the formation of smooth and homogeneous thin layers, high stability, and strong adhesion to the electrode surface^33,48,49^also the polymer films are not only contribute to the formation of additional active sites available for target interaction with analytes but also enhance the sensor’s sensitivity^50^. Very Recent examples of amino acids include polyserine (SER)^48^, polymethionine^50^, poly(L-lysine)^33^, polyglutamic acid^29^, Poly (L-Cysteine)^51^.

Hippuric acid, also known as N-benzoyl glycine, is an amino acid derivative formed through the conjugation of benzoic acid with glycine in the liver^52^and is excreted in the urine of humans and other mammals^53^. Hippuric acid with its unique structure has shown a promising applicability as an electropolymerizable molecule. Consequently, we here in present for the first time, the application of electropolymerized hippuric acid as an electrode modifier for recycled battery graphite in combination with multiwalled carbon nanotubes, poly (HA)/MWCNTs/BGE sensor as a sensing platform for the determination of SER. The analytical performance of the modified electrode for quantification of SER in the presence of tryptophan and in biological fluids such as human blood serum, was elaborated. Furthermore, the greenness of the proposed sensor was evaluated using the National Environmental Index (NEMI), Analytical Greenness Metric (AGREE), the modified NEMI by Raynie and Driver, the Green Analytical Procedure Index (GAPI), and the Analytical Eco-Scale, to assess the eco-friendliness and environmental sustainability of the presented approach.

## Experimental

### Reagents and materials

All chemical reagents in this study were of analytical grade and used without further purification. Serotonin (95%), (SER), L-tryptophan (≥ 98%) (TRP), Hippuric acid (98%), potassium dihydrogen phosphate anhydrous, dipotassium hydrogen orthophosphate, phosphoric acid (85%), sodium hydroxide, Nafion (5%), N, N dimethylformamide (DMF) (99.8%), Isopropanol (99.9%), potassium ferricyanide, potassium ferrocyanide trihydrate and potassium chloride, were purchased from Sigma-Aldrich, Germany.

The selectivity studies were performed using DL-Norepinephrine hydrochloride (≥ 97%) (NE), D-glucose (≥ 99.5%), Folic acid (FA), Ascorbic acid (AA) and Uric acid (UA) (Sigma-Aldrich, Germany). Multiwalled carbon nanotubes (MWCNT) with a diameter range of (10–20 nm) and (5–15) µm length were purchased from Tokyo chemical industries, Japan. All aqueous solutions used throughout this work were prepared in Milli-Q water purified in pure lab UHQ (ELGA, United Kingdom).

### Apparatus

Palm-SensTrace 5 EIS potentiostat/galvanostat (Palmsens BV, Houten Netherlands) controlled by PSTrace version 5.0 software was used for carrying out all electrochemical measurements. The three-electrode system consisted of an Ag/ AgCl electrode as a reference electrode, 1.0 mm diameter platinum wire as a counter electrode and Battery graphite electrode as a working electrode. A digital 3510 pH meter (Jenway Instruments, Staffordshire, UK) using a combination glass electrode was used for pH measurements. Scanning Electron Microscopic analysis (SEM) was conducted using (Quanta-FEG250FEI high resolution SEM), While Surface topographic characterization was performed using a 5600Ls atomic force microscopy (AFM) unit, (Agilent Technologies, Inc., Santa Clara, CA, USA) and (JEM-2100 F, JEOL, Japan) operating at 200 kV was used for Transmission Electron Microscopy (TEM ) investigation. FT-IR experiments were carried using A Nicolet iS50 FT-IR Spectrometer (Thermo Fisher Scientific, Germany).-IR experiments were carried out in the Microanalytical.

### Preparation of solutions

Stock solutions of SER or TRP (10 mM) and other interfering biological samples (norepinephrine hydrochloride (NE), D-glucose, Folic acid (FA), Ascorbic acid (AA) and Uric acid (UA) (1mM) were prepared and lower concentrations were freshly diluted using phosphate buffer solution (PBS) pH 7.0 for each experiment.

0.1 M PBS was prepared by dissolving suitable amounts of K_2_HPO_4_ and KH_2_PO_4_ in ultra-pure water and adjusting pH with 0.1 M NaOH or H_3_PO_4_ as required. 1mM Hippuric acid (HA) was prepared by dissolving the calculated amount in 0.1 M PBS pH 7.4, 10 mg of MWCNTs was added to 10mL DMF followed by ultrasonication for 2 h until a homogenous suspension of MWCNTs (1 mg/ml) was obtained.

Serum samples, provided by a public clinical laboratory from anonymous volunteers, were diluted 100 times in 0.1 M PBS pH 7.0 without any other pretreatment and the standard addition method was applied for analysis at different spiked concentrations.

### Preparation of the working electrode

The working electrode was made from the graphite bar extracted from zinc/carbon waste batteries (size AAA). The 3 mm diameter graphite rod was thoroughly cleaned using 0.5 M H_2_SO_**4**_ to eliminate any traces of the zinc paste adhered to its surface then was rinsed with water and resoaked in ethanol for 5 min to further remove any impurities. The cleaned graphite rod was then inserted into 3 mm diameter and 5 cm length dielectric PTFE (Polytetrafluoro-ethylene) heat shrink tubing wire and placed in an oven at 80 °C for 30 min to ensure full coverage and adherence of the shrink to the electrode body. 0.5 cm length from one end of the electrode body was left uncovered to be used for connection while the other end was mechanically polished using rough emery paper grade P130, followed by grades P1000 and P2000 to assure the smoothness of the electrode body until achieving a mirror-like polished surface.

### Fabrication of poly (HA)/MWCNTs/BGE

Prior to modification, the bare carbon graphite electrode was cleaned by polishing on a paper for several times till a mirror-like surface was attained, washed thoroughly with double distilled water followed by sonication with ultra-pure water for 3 min to remove any particles adhered to its surface, from previous measurments, then the electrochemical activation of the electrode was performed by cyclic voltammetric scanning in 0.1 M NaOH within a potential window from − 0.5 V to 1 V at scan rate 50 mV/s until stable CV peaks were obtained.

The modified sensor was then developed through drop casting of 7µL of MWCNTs (1 mg/mL) on the cleaned BGE surface and left to dry at room temperature, then 5 µL of 0.5% (v/v) Nafion solution diluted in isopropanol were drop-casted as conductive binder that stabilizes the multiwalled carbon nanotubes particles on the surface of the working electrode to yield the MWCNTs/BGE sensor. Finally poly (HA)/MWCNTs/BGE was fabricated by electropolymerization of 1mM Hippuric acid in 0.1 M PBS pH 7.4 from (−1 V to 1.5 V) at a scan rate of 0.1 V/s for different number of cycles, then the electrode was carefully washed with distilled water to remove any physically adsorbed hippuric acid and kept to dry at room temperature.

### Experimental measurements of SER

The electrochemical sensing of SER using poly (HA)/MWCNTs/BGE, was performed employing differential pulse voltammetric (DPV) measurements at a potential window from − 0.3 V to 0.6 V, step potential 0.01 V, pulse potential 0.2 V, pulse time 0.05 s and scan rate 0.05 V/s. The measurements were done in phosphate buffer solution (PBS) pH 7.0. The electrochemical characterization of the electrode was investigated in 0.1 M KCl solution as a supporting electrolyte containing 2.5 mM [Fe(CN)_6_] ^3−/4−^, used as an active probe for the characterization using electrochemical impedance spectroscopy (EIS) at a constant potential of 0.2 V, frequency range (0.1 Hz 100 kHz) and amplitude 0.01 V. All electrochemical measurements were carried out at room temperature.

## Results and discussion

### Optimization of the electrode’s modification steps

**Fig. 1:**
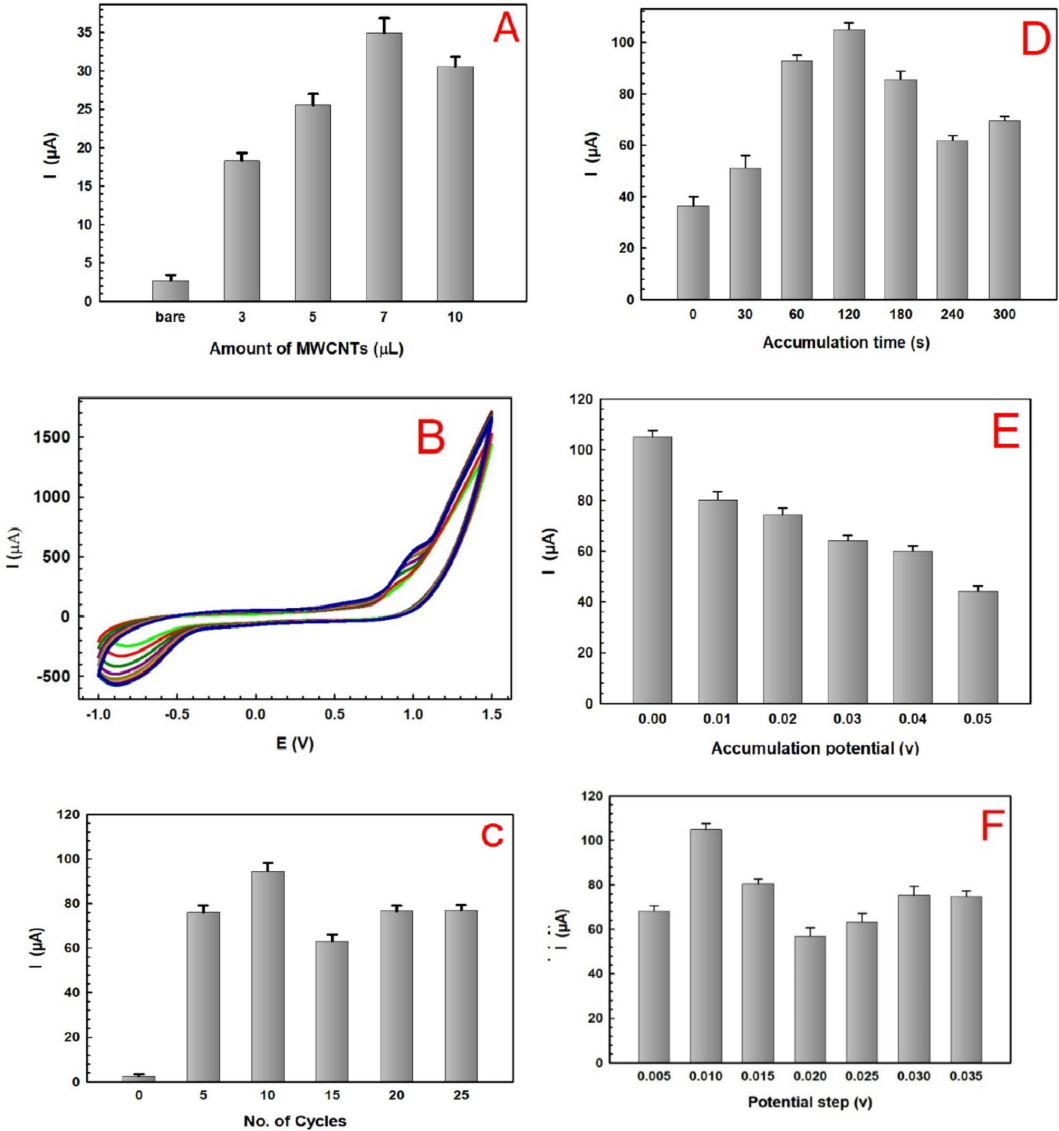
(**A**) Effect of the amount of MWCNTs. (**B**) CV of ten cycles electropolymerization of 1mM Hippuric acid in potential range from − 1 to 1.5 V and at a scan rate 100 mV/s. (**C**) Effect of number scan cycles of the electropolymerization of Hippuric acid on the potential response towards 10 µM SER in 0.1 M PBS pH 7. (**D**) Effect of Effect of accumulation time. (**E**) Effect of accumulation potential and (**F**) Effect of potential step towards 10 µM SER in 0.1 M PBS pH 7.

Different fabrication parameters are found to affect the response performance of the presented sensor, including: the content of MWCNTs, concentration of hippuric acid used for polymerization and the number of polymerization cycles besides other DPV operational parameters as accumulation time, accumulation potential and potential step, that need to be investigated to reach the optimum modification conditions, as shown in Fig. 1.

### Effect of the MWCNTs content

The amount of MWCNTs is an important factor to be optimized due to its effect on the performance of the BGE electrode. Different amounts of MWCNTs ranging from 3 to10 µL were drop-casted on the surface of the bare BGE, and their impact on the response towards 10 µM SER was tracked using DPV. As clear in Fig. 1A, a current response of 17.28 µA was attained after 3 µL compared to bare BGE which showed a very small current response of 3 µA. The response was increased to reach 24.26, 36.78 µA after drop-casting and 7 µL, respectively, further increase in the amount of MWCNTs to 10 µL has resulted in a decrease in the current response to become 29.28 µA. The increase in the amount of MWCNTs can lead to the aggregation of the nanotubes, forming a thicker film on the electrode’s surface, resulting in poor dispersion of the MWCNTs and in turn a decrease in the electrical conductivity as a consquence^41,54^, thus, may affect negatively on the electrochemical performance of the sensor. Accordingly, 7 µL was taken as an optimum amount of MWCNTs to cast on the BGE followed by drop-casting 5µL of 0.5% (v/v) Nafion solution diluted with isopropanol, to cover MWCNTs layer to assure its stability and render the MWCNT/BGE sensor.

### Electropolymerization of hippuric acid

Polyhippuric acid, poly (HA), was fabricated by the electropolymerization of hippuric acid over MWCNTs/BGE surface using cyclic voltammetry within the potential window − 1.0 to 1.5 V and at a scan rate 100 mV s^−1^ at different number of polymerization cycles ranging from 5 to 25 cycles. The CVs recorded after 10 cycles of electropolymerization as shown in Fig. 1B, indicating the formation of a polymeric layer of poly (HA) on the surface of MWCNTs/BGE.

Different concentrations of hippuric acid, ranging from 10^−4^ to 10^−2^ M in PBS pH 7.4, were tested for the electropolymerization. The change in concentrations was shown to have no significant change in response towards 10 µM SER, consequently 1mM was chosen for all further optimizations. The suggested mechanism for electropolymerization can be presented as:
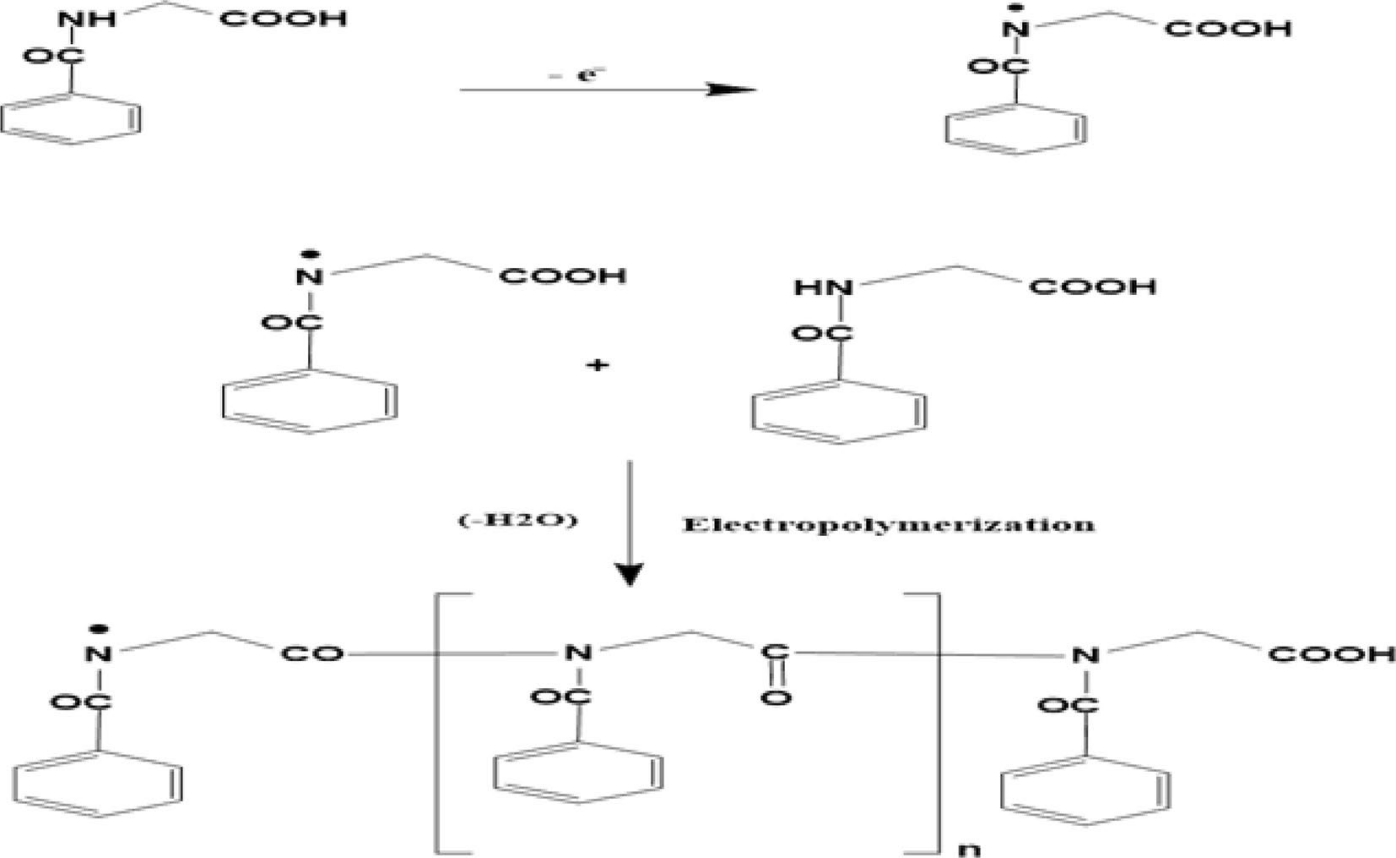


This approach agrees with the previously reported electropolymerization conditions for other amino acids, example of which, DL-methionine which was electropolymerized on AuNPs/GCE from − 0.8 V to 1.5 V at scan rate 100 mV s^−1^in detection of the pharmaceutical compound paroxetine^50^, DL-serine was electropolymerized on a fMWCNTs & rGO-modified GCE in a potential range from − 1.1 to 2.5 V at scan rate 50 mV s^−1^for determination of agricultural residues of Kasugamycin antibiotic^48^ .

The thickness and conductivity of the conducting polymeric film deposited over MWCNTs/BGE are influenced by changing the number of polymerization cycles ranging from 5 to 25 cycles as shown in Fig. 1C. The peak current was found to increase from 78.86 to 95.96 µA after 5 and 10 cycles, respectively, further increase in the number of cycles from 15 to 25 cycles has resulted in a decrease in the current response to 63.40 and 77.66 µA, respectively. This can be interpreted as, a limited number of cycles resulted in an unstable layer that could be removed from the electrode surface, while the peak current decreased after 10 cycles due to the formation a compact insulating layer on the electrode surface and the process of diffusion becomes more difficult^32^which indicates an increase in the polymeric matrix thickness that alter the conductivity of the film^50^. Thus, the optimum number of cycles was adjusted to be 10 cycles, which were sufficient enough to produce the maximum oxidation current of SER.

### Effect of DPV operational parameters

The effect of accumulation time (t_acc_) on the peak current of 10 µM SER was studied from (0–300 s), as shown in Fig. 1D. The peak current was found to increase from 36.33 to 92.72 µA after 0 and 60s, respectively; a current response of 105.61 µA was attained after 120 s then decreased to reach 85.66 to 70.35 µA after 180 and 300s, respectively. This indicates that the maximum oxidation peak current of SER was observed at 120 s and accordingly was chosen to be the optimum accumulation time for subsequent experiments .

The accumulation potential (E_acc_.) was also studied from (0–0.05 V), as given in Fig. 1E, increasing the accumulation potential resulted in decreasing the response of poly (HA)/MWCNTs/BGE sensor, thus zero E_acc_. was applied during the measurements of SER. The potential step (E_s_) effect was tested from (0.005 to 0.035 V), as given in Fig. 1F, on increasing E_s_ from 0.005 to 0.01 V, the current response was found to be 66.65 µA and 105.61 µA, respectively resulting in a better response but on subsequent increase of E_s_ from (0.02 to 0.035 V) resulted in a negative impact on the current response, declining to 59.64 µA and 78.04 µA, respectively, and also resulted in the deformation of the recorded peaks, thus 0.01 V was applied as the optimum potential step.

### Effect of pH

The influence of pH on the electrochemical response of poly (HA)/MWCNTs/BGE for the oxidation of SER was performed in 0.1 M PBS of pH (5.5–8.0) using differential pulse voltammetry (DPV).

**Fig. 2:**
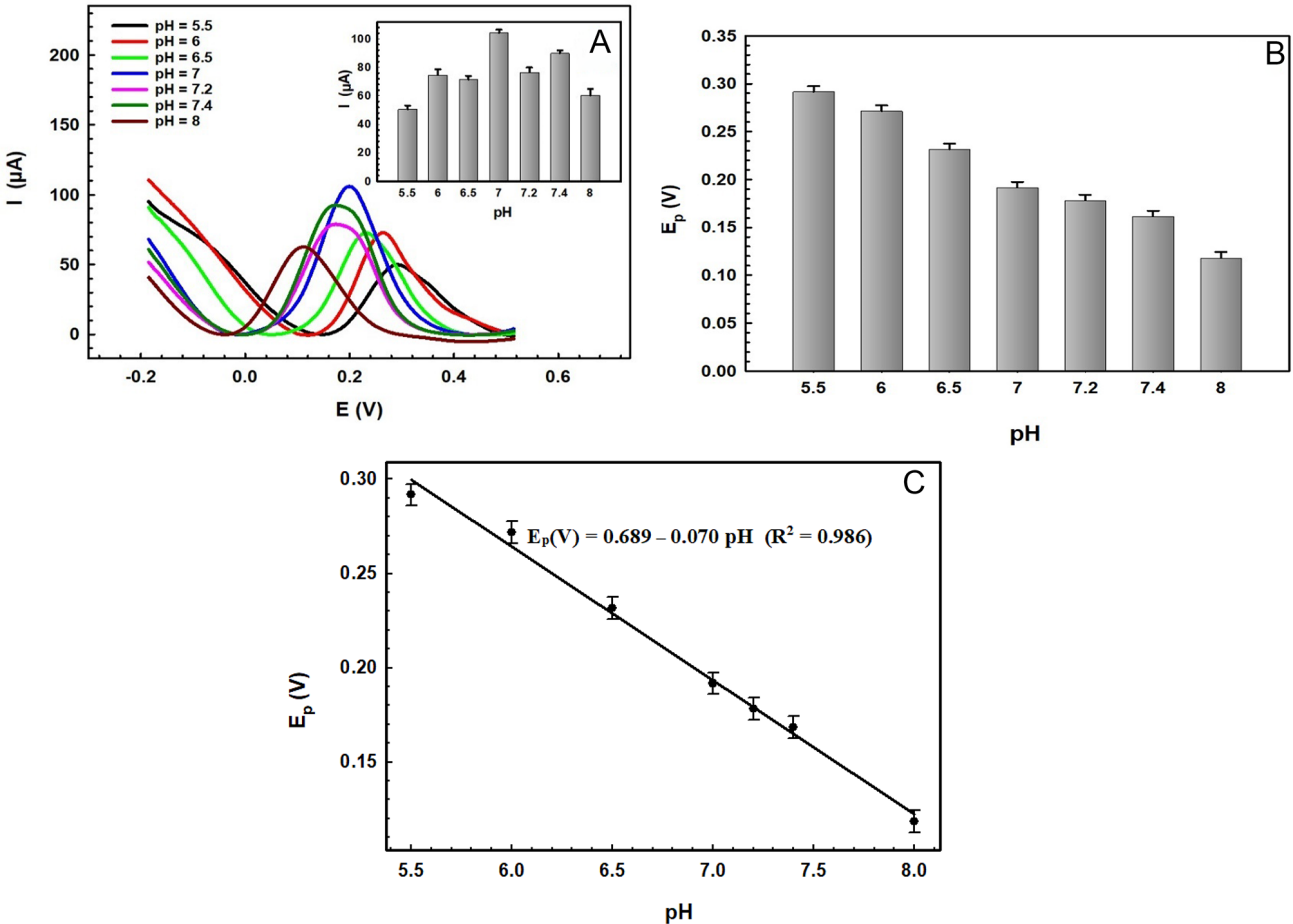
(**A**) DPV for oxidation of 10 µM SER in 0.1 M PBS at different pH in range from 5.5 to 8 (Inset: represents the effect of pH medium on peak current). (**B**) Effect of pH medium on peak potential. (**C**) The relation between peak potential and pH for the tested pH values.

SER has two pKa values, the first pKa value is 9.31 corresponding to the hydroxyl group and the second pKa value is 10 corresponding to the amino group, respectively^55^. According to the correlation between current and pH shown in Fig. 2A, the oxidation peak current was found to increase as the pH value increased up to pH 7.0, and after which the peak current was found to decrease due to the deviation from the neutral condition. The results can be explained to be as a result of suppression of SER oxidation at low pH due to the high proton concentration and while at a pH above 8.5, SER itself might undergo polymerization which reduces its concentration^23,56^, thus, the maximum peak current was found at pH 7.0 and accordingly, was chosen as the optimum pH for subsequent experiments which agrees with similar observations previously reported literature^23,31,57^.

The graph representing the correlation between E_p_ and pH, shown in Fig. 2B, indicates that the peak potential (E_p_) of SER was observed to shift negatively with increasing the pH value and no reduction peak was observed in the cathodic part of the voltammogram confirming that the process of SER interaction at the poly (HA)/MWCNTs/BGE is a completely irreversible oxidation process^2,58^.

Figure 2C, represent a linear correlation between Ep and pH and on applying to Philip’s Rieger relationship represented as:

$$E_p (V)=k-(0.059 \frac{y}{n}) pH$$

where k: the intercept of the linear relation between E_p_and pH, y: number of protons and n: number of electrons during at the electrochemical oxidation reaction^59–61^. It is clear that the pH value presented a linear relationship with E_p_ indicated as:

$$E_p(V)=0.689-0.070\; pH, R^2=0.986$$

The slope of the line given in figure of E_p_/pH was found to be 0.070 V pH^−1^, which is comparable with the theoretical value of 0.059, and in accordance, it can be depicted that the oxidation process of SER requires equal numbers of protons and electrons^1,58,62^which agrees with the suggested electro-oxidation reaction mechanism for SER involving 2-electrons and 2-protons, as shown in the following equation^1,2,31,32,62^:.
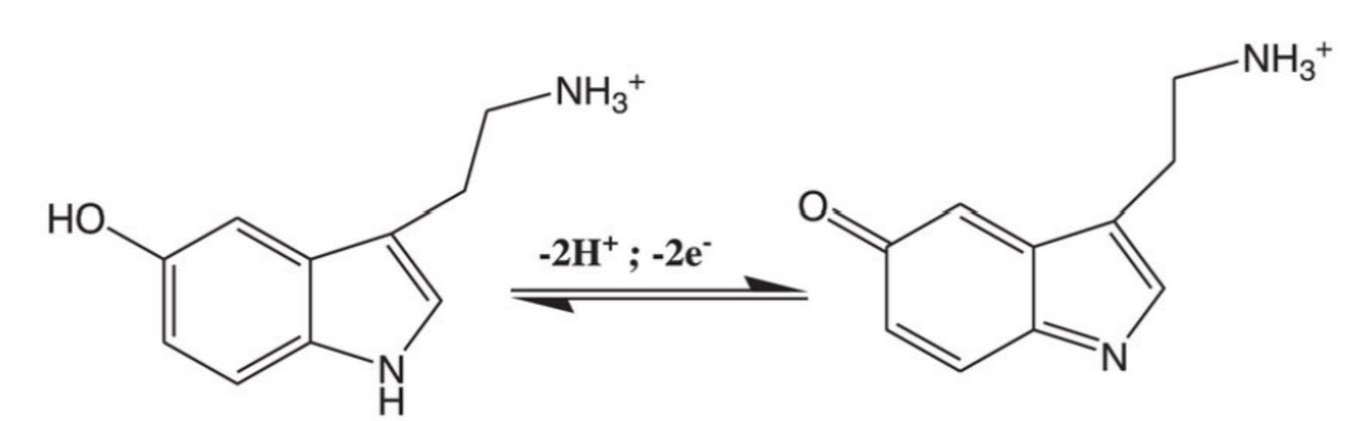


### Surface morphology, Topography and FTIR of the modified sensor

**Fig. 3:**
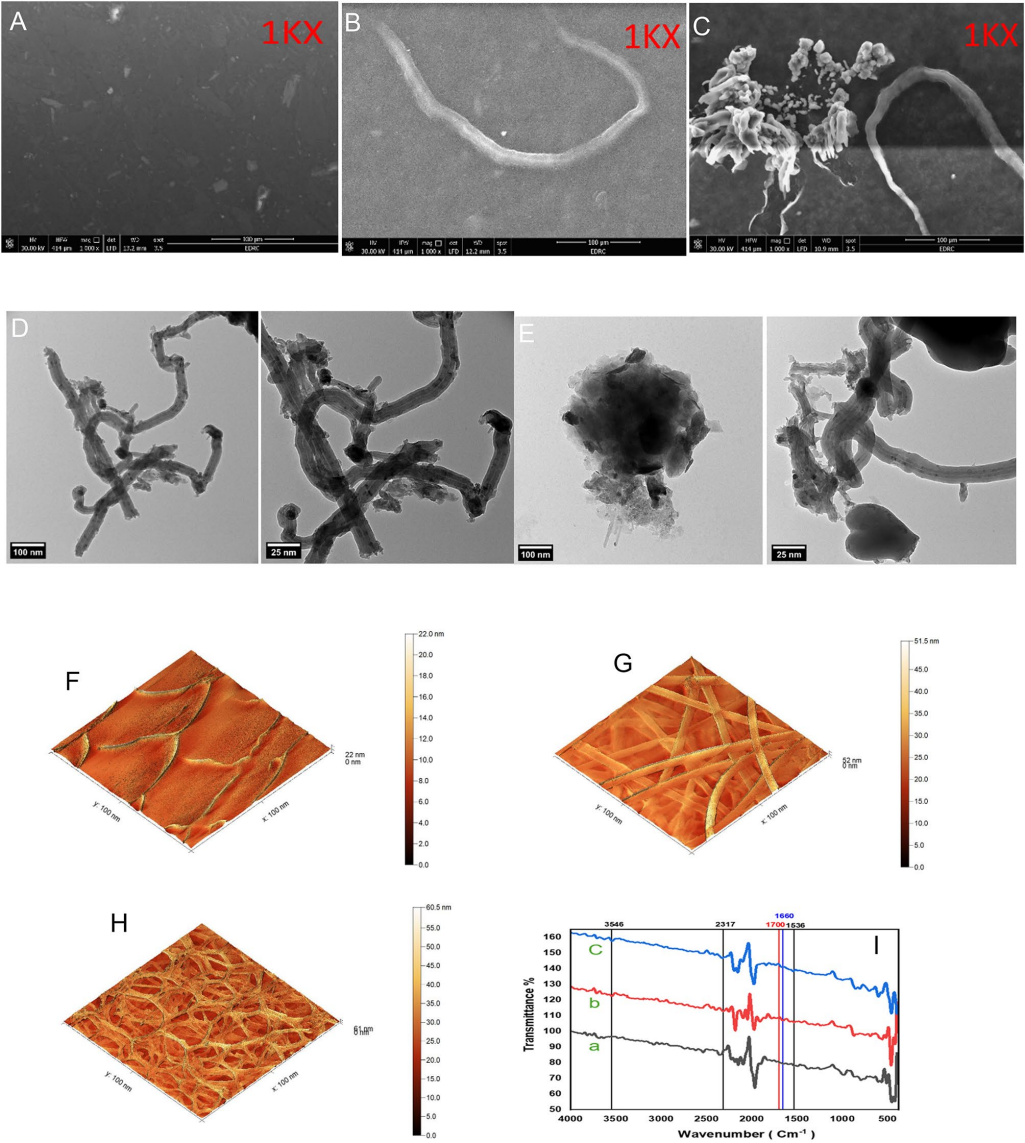
SEM images of Bare BGE (**A**), MWCNTs/BGE (**B**), poly (HA)/MWCNTs/BGE (**C)** at magnification 1KX. TEM images of MWCNTs/BGE (**D**), poly (HA)/MWCNTs/BGE (**E**). AFM images of Bare BGE (**F**), MWCNTs/BGE (**G**), poly (HA)/MWCNTs/BGE (**H**) and FTIR for bare (a), MWCNTs/BGE (b) and poly (HA)/MWCNTs/BGE (c).

Scanning electron microscope (SEM) was used to study the surface morphologies of bare BGE, MWCNTs/BGE, poly (HA)/MWCNTs/BGE, samples were fixed at working distance 13.2 mm, an excitation voltage of 30 KV and at magnification 1KX. Figure 3 A, shows a smooth and clean surface of bare BGE, while Fig. 3B shows the electrode’s surface after modification with MWCNTs. Few MWCNTs are found to be distributed on the surface of BGE with ribbon-like tubular structures which may enhance the active surface area of the electrode as result of using small size of MWCNTs particles (10–20 nm), Finally, Fig. 3C representing the modified poly (HA)/MWCNTs/BGE, shows that the polymer was successfully synthesized on the surface of MWCNTs/BGE resulting in a modified surface morphology that appears to be compact and agglomerated.

Transmission electron microscopy (TEM) analysis was also applied to identify the morphology of MWCNTs/BGE, and poly (HA)/MWCNTs/BGE at magnification of 100 nm and 25 nm. Figure 3D shows TEM image of MWCNTs with their tubular / hollow tube structures, while Fig. 3E, representing poly (HA)/MWCNTs/BGE, clearly shows the formation of a polymeric layer covering the MWCNTs .

Atomic force microscopy (AFM) was also used to investigate morphology and topography of the poly (HA)/MWCNTs/BGE) with contact mode and 100 nm × 100 nm scan area for bare BGE, MWCNTs/BGE and poly (HA)/MWCNTs/BGE. Figure 3 F shows the bare surface of BGE, as a smooth surface with no pores and exhibiting root mean square (RMS) roughness of 1.43 nm. Figure 3G indicates the deposition a thin film layer of MWCNTs and the RMS roughness for MWCNTs/BGE was increased to become 4.11 nm. While in Fig. 3H representing poly (HA)/MWCNTs/BGE, a further increase in RMS roughness was noticed reaching 5.91 nm indicating the successful electropolymerization of hippuric acid and formation of the compact polymeric film from poly (HA) on the surface of MWCNTs/BGE.

The FTIR spectrum of MWCNTs (b), Fig. 3I, shows characteristic peaks at 1660, 2317, and 3546 cm^−1^, which are absent in the bare BGE (a). The peak at 1660 cm^−1^ confirms the presence C = C stretching vibration, while the peak at 2317 cm^−1^ corresponds to the graphitic component of MWCNTs, and the peaks at 3546 cm^−1^ can be attributed to O–H stretching vibration of water vapour which may be adsorbed by MWCNTs surface. The FTIR spectrum of poly (HA) deposited on MWCNTs/BGE (c) was found to be similar to that MWCNTs surface and also displays some peaks with a small shifts in the wavenumbers at 1536, 2327, and 3537 cm^−1^, these characteristic peaks can be attributed to the presence of MWCNTs while the peak at 1700 cm^−1^is attributed to C = O stretching vibration^63–65^.

No new peaks were recorded which indicates a low probability of new covalent bonding between the carbon nanotube and the polymer^66,67^. The small shifts in the wavenumbers in the FTIIR spectrum suggests a limited change in the chemical environment of the polymer formed on the surface of carbon nanotubes. It was also noted that the broad peak associated with the O-H stretching vibration of carboxylic group in hippuric acid is absent from the spectrum indicating the involvement of O-H group in the electropolymerization process.

### Electrochemical characterization of the modified electrode

The modified sensor was characterized using cyclic voltammetry (CV) and electrochemical impedance spectroscopy (EIS) that are capable of tracking the mechanism of electrochemical interaction its impact on the conductivity and electron transfer at the modified electrode surface.

**Fig. 4:**
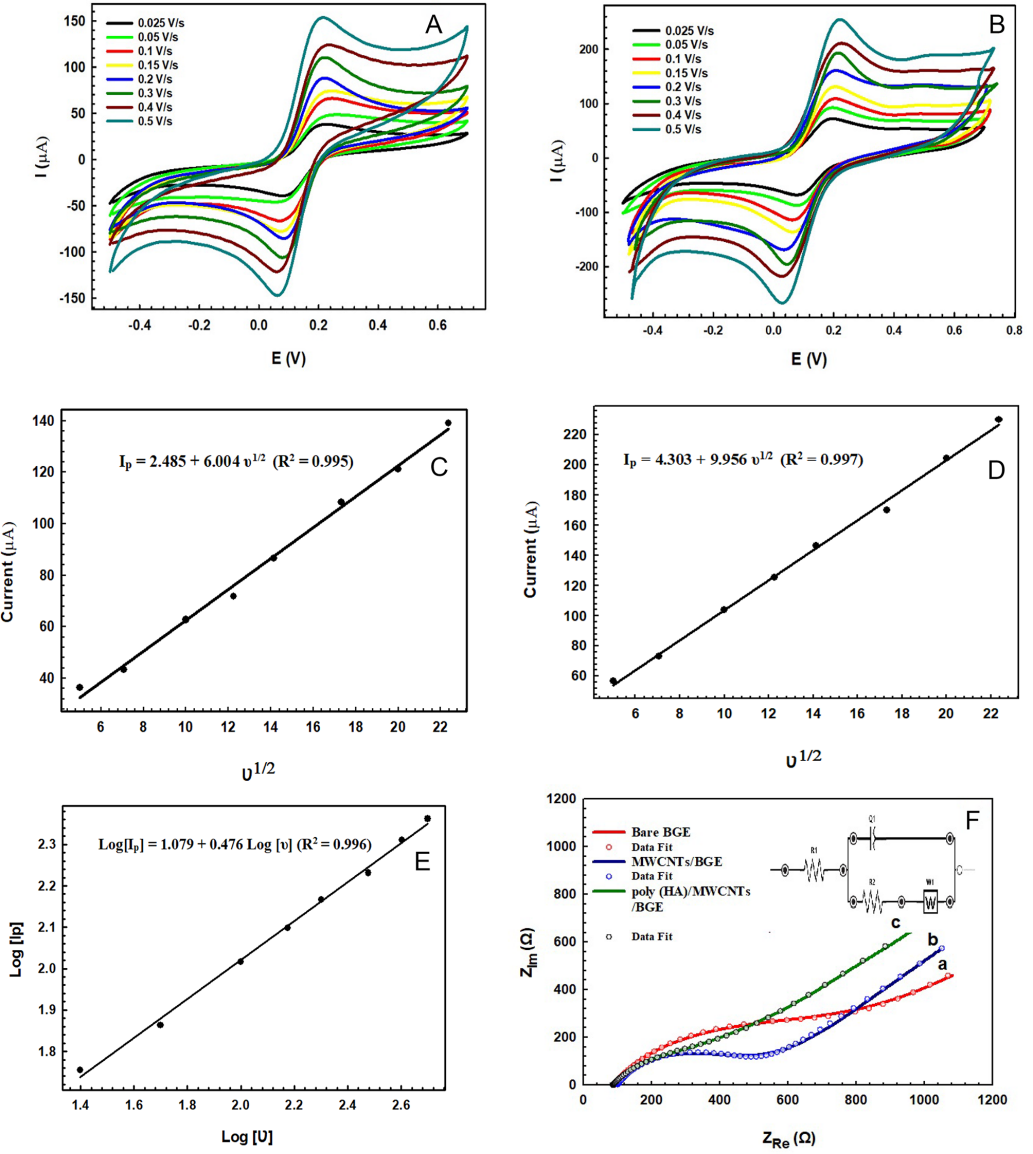
(**A**) Cyclic voltammogram in 2.5 mM [Fe(CN)_6_] ^3−/4−^ in the presence of 0.1 M KCl at different scan rate from 25 to 500 mV/s at Bare BGE and (**B**) at poly (HA)/MWCNTs/BGE sensors. Linear relation between current and square root of scan rate for; (**C**) Bare BGE, (**D**) poly (HA)/MWCNTs/BGE sensors and (**E**) Linear relation between the logarithm of scan rate and logarithm current of the anodic peak for poly (HA)/MWCNTs/BGE biosensor. (**F**) Nyquist plots of EIS for bare BGE (a), MWCNTs/BGE (b), poly (HA)/MWCNTs/BGE (c) in 2.5 mM [Fe(CN)_6_] ^3−/4−^ dissolved in 0.1 M KCl solution at potential 0.2 V, frequency range (0.1 Hz–100 kHz) and amplitude 0.01 V.

Cyclic voltammetry (CV) measurements were utilized to examine the influence of varying scan rates on the response of anodic and cathodic peak currents. Figure 4A and B show CVs for both bare BGE, poly(HA)/MWCNTs/BGE at different scan rate in the range of 25 to 500 mV/s carried out in 2.5 mM [Fe(CN)_6_]^3−/4−^prepared in 0.1 M KCl. It is clear that the peak current was found to increase on increasing the scan rate from 25 to 500 mV/s and the response of peak current of poly (HA)/MWCNTs/BGE is higher than that of bare BGE, this enhancement can be attributed to the increase in the active surface area of the electrode in the presence of MWCNTs^68,69^and the synergetic effect of poly(HA) and MWCNTs on BGE which enhances the electron transfer^48,70^.

When the anodic (І_p.a._) and cathodic (І_pc_) peak current values were plotted against the square root of scan rate (υ ^1/2^), as shown in Fig. 4C and D for bare BGE and poly(HA)/MWCNTs/BGE, respectively, a linear relationship with two regression equations was attained^50,59,71^:

$$I_{pa} = 2.485+6.004v^\frac{1}{2}\; with\; (R2=0.995), and$$

$$I_{pc}=4.303+9.956v^ \frac{1}{2} \;with\; (R2=0.997)v$$

The effective electroactive surface area (A_eff_) of the bare BGE, modified poly(HA)/MWCNTs/BGE have been calculated by Randles-Sevcik Eqs^5,39,72^..

$$I_{pa}=(2.69\times10^5)n^\frac{3}{2}A(D_o)^\frac{1}{2}C_ov^{1}{2}$$

where I_p.a._ is the anodic peak current (µA), n is the number of electrons (*n*= 1), A is the electroactive surface area (cm^2^ ), D is the diffusion coefficient of [Fe(CN)_6_]^3−/4−^ (7.6 × 10^−6^cm^2^ s ^−1^ ), C_o_ is the concentration of the Fe(CN)_6_]^3−/4−^(2.5 µM), υ is the scan rate (V/s)^73–75^.

The A_eff_of bare BGE and poly (HA)/MWCNTs/BGE were calculated to 0.1019 cm^2^, and 0.1752 cm^2^, respectively. The increase in the surface area of the modified electrode compared to the bare electrode can be correlated the enhancement effect, conductivity, electron transfer at the modified electrode were significantly accelerated compared to bare BGE^51^, providing more active sites for the electrooxidation of SER as a result of the surface modification.

The linear relationship between logarithm of the scan rate and logarithm of the anodic peak current, is shown in Fig. 4E, with a regression equation:

$$Log[I_p]=1.079+0.476\; log [v] \;with \;R^2 = (0.996)$$

and the obtained slope was found to be 0.476 which is quite close to the theoretical slope value of 0.5. This behaviour suggests that the oxidation of SER at the modified poly (HA)/MWCNTs/BGE electrode is diffusion controlled process^70,71,76^.

Electrochemical impedance spectroscopy (EIS) was also employed to investigate the changes of the interfacial properties of the surface of modified electrodes^77,78^. The charge transfer resistance of the electrode (R_ct_) was obtained through fitting with Randle’s equivalent circuit as represented in (inset of Fig. 4F )^50^.

The Nyquist plot of the EIS was shown to include a semicircular part observed at higher frequencies representing the charge transfer resistance (R_ct_) and while the linear part observed at lower frequencies represents the diffusion process^78^. All studies were carried out in 2.5 mM [Fe(CN)_6_]^3−/4−^ containing 0.1 M KCl solution, as a chemical probe, as represented in Fig. 4F indicating the Nyquist plots for the bare BGE (curve a), MWCNTs/BGE (curve b), poly (HA)/MWCNTs/BGE (curve c).

The fitted Randle’s equivalent charge transfer resistance (R_ct_) at the bare BGE, MWCNTs/BGE, poly (HA)/MWCNTs/BGE were found to be 716.3, 401.6 and 376.6 Ω, respectively. The bare BGE exhibited the highest R_ct_ value due to its lower conductivity, however, upon modification of BGE the R_ct_value was decreased and the lowest value was observed for poly (HA)/MWCNTs/BGE when compared to other electrodes due to the synergetic effect of poly (HA) and MWCNTs on BGE which enhances the electron transfer process and improves the electrocatalytic activity^48^. Thus, this sensor has promising applicability for use in electrochemical detection of SER.

### Interference studies

The selectivity of the poly (HA)/MWCNTs/BGE was investigated by recording DPV for solutions containing 10 µM SER mixed with varying concentration ratios 1:1, 1:5 and 1:7 of the tested major possibly interfering biological substances namely, ascorbic acid, uric acid, norepinephrine, folic acid and glucose, as shown in Table 1. It is clear that there was no significant difference (± 5% ) in the peak current of SER when mixed with tested interfering substance at different ratios, with relative standard deviation (RSD) less than ± 5% ( *n* = 3), indicating that the poly (HA)/MWCNTs/BGE sensor is highly selective towards the determination of SER, and its efficiency and applicability in complex matrix.

### Method validation

**Fig. 5:**
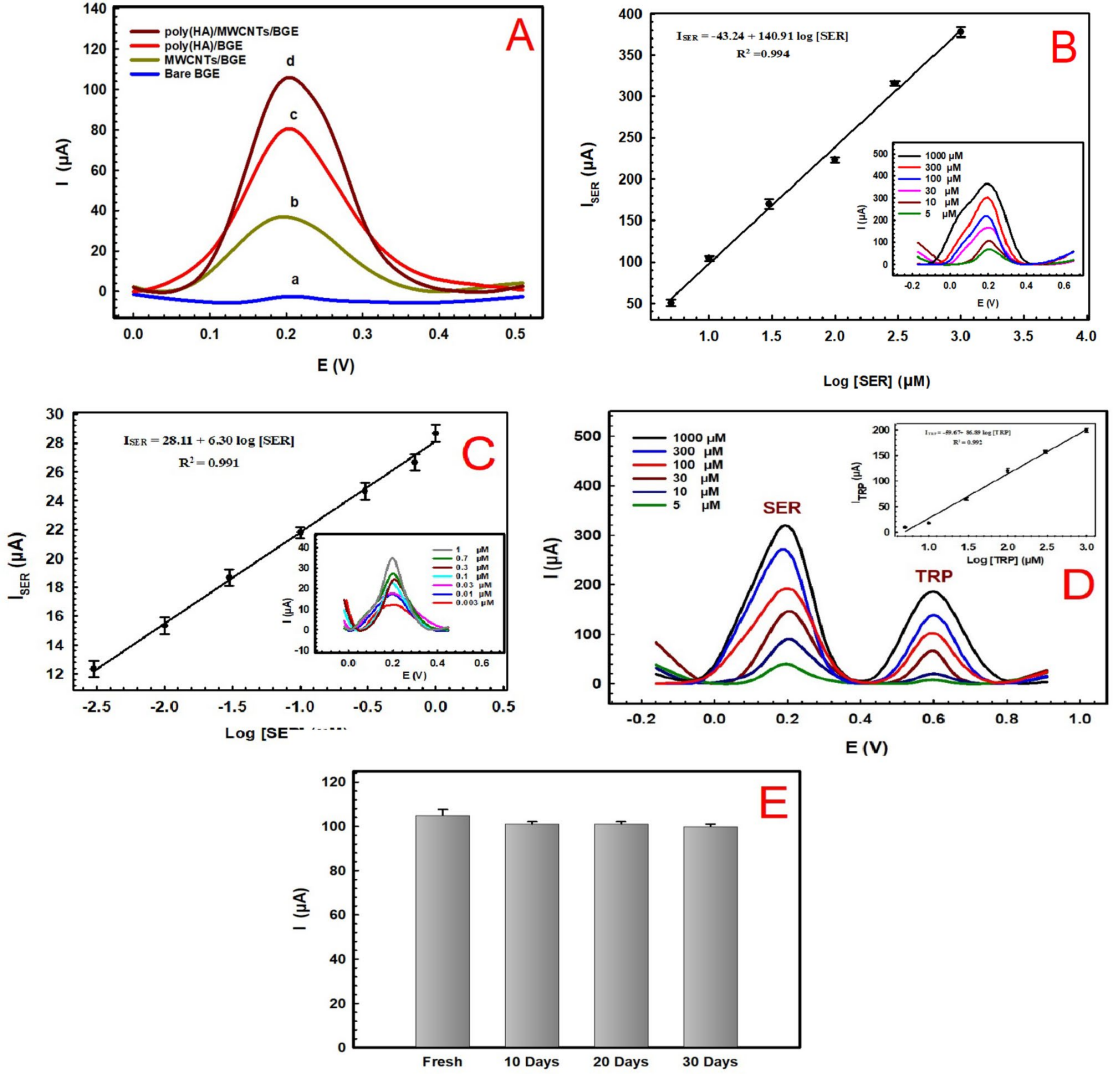
(**A**) DPV of 10 µM SER in PBS pH 7 at bare BGE (a), MWCNTs/BGE (b), poly (HA)/BGE (c), poly (HA)/MWCNTs/BGE (d) at optimum working conditions. (**B**) Calibration curve of poly(HA)/MWCNTs/BGE for SER for concentrations 5.00 to 1.00 × 10^3^ µM and (**C**) 3.00 × 10^−3^ to 1.00 µM SER in 0.1 M PBS at pH 7. (Insets of Fig. 5B and C represent the DPV response of two concentration ranges 5.00 to 1.00 × 10^3^ µM, and 3.00 × 10^−3^ to 1.00 µM, respectively). (**D**) DPVs of poly (HA)/MWCNTs/BGE in 0.1 M PBS pH 7 containing different concentration of SER and TRP from 5 to 1000 µM (Inset: represents the Calibration curve for TRP). (**E**) Stability of modified poly (HA)/MWCNTs/BGE sensor.

Under the optimized conditions, the modified sensor exhibited high sensitivity towards SER, as shown in Fig. 5A, representing the DPVs of the oxidation of 10 µM SER prepared in PBS pH 7.0 at different modified sensor’s stages: bare BGE, MWCNTs/BGE, poly (HA)/BGE, poly (HA)/MWCNTs/BGE in a potential window from (−0.3 V to 0.6 V).

The bare BGE, (Curve a), showed a non-significant oxidation peak at approximately 0.2 V, as for MWCNTs/BGE, (Curve b), an enhanced oxidation peak current was obtained due to the increase in the active surface area of the sensor in the presence of MWCNT compared to the bare surface^69^. While, on using poly (HA)/BGE, (Curve c), the oxidation peak was higher than that of bare BGE, confirming the formation of a conducting film of poly(HA) in a stable coating on the electrode with high conductivity consequently enhancing the electron mobility within the conducting polymer. The highest current response was recorded for the oxidation of SER for the poly(HA)/MWCNTs/BGE sensor as shown in (Curve d) due to the synergistic effect of MWCNTs and conducting film of poly(HA) improved the oxidation of peak current of SER, thus enhances the efficiency, conductivity and sensitivity of the electrode.

The DPV response of SER on the surface of poly (HA)/MWCNTs/BGE sensor exhibits two linear concentration ranges. The peak current of SER increased linearly with increase in its concentration from 3.00 × 10^−3^ to 1.00 × 10^3^ µM. Figure 5B shows the calibration curve for the first linearity range from 5.00 to 1.00 × 10^3^ µM (0.88–176.22 µg/ml) with regression equation:

$$ISER=-43.24+140.91\; log[SER] \;and\; (R^2=0.994)$$

while Fig. 5C shows the second linearity range from 3.00 × 10^−3^ to 1.00 µM (5.29 × 10^−4^ – 0.18 µg/ml) with and equation presented as:

$$ISER=28.11+6.30\; log[SER] \;and\; (R^2=0.991)$$

**Table 1:** Interference studies for the determination of SER in the presence of other biological substance.

Interferent	Structure	Conc. ratio (SER: Interferent)	Recovery (%)	RSD (%)
Ascorbic acid		1:1	102.53%	2.33%
		1:5	104.12%	1.05%
		1:7	103.80%	2.42%
Uric acid		1:1	97.46%	3.43%
		1:5	100.31%	2.90%
		1:7	96.82%	3.70%
Norepinephrine		1:1	95.23%	4.00%
		1:5	105.71%	1.56%
		1:7	94.87%	1.54%
Folic acid		1:1	96.19%	1.98%
		1:5	105.71%	1.56%
		1:7	98.73%	2.42%
Glucose		1:1	103.17%	3.73%
		1:5	105.07%	1.38%
		1:7	100.00%	4.36%

The limit of detection (LOD) calculated based on (S/*N* = 3) and limit of quantification (LOQ) calculated based on (S/*N* = 10 ) were found to 0.06 × 10^−2^ µM (1.06 × 10^−4^ µg/ml) and 2.00 × 10^−3^ µM (3.52 × 10^−4^ µg/ml), respectively. The linear range and LOD of the poly (HA)/MWCNTs/BGE were found to be comparable with other previously reported for the determination of SER as shown in Table 2. Previously reported modified sensors exhibited LOD ranging from 1.90 × 10^−3^ to 5.70 µM, this indicates that the proposed sensor offers a better limit of detection and dynamic range when compared to other reported modified electrodes. The enhanced performance of the poly (HA)/MWCNTs/BGE sensor can be attributed to its effective improvement in electron transfer kinetic, high electrocatalytic activity, and high sensitivity, making it an a promising tool for SER analysis, besides being based on a recycled waste which is an important advantage when compared to other previously reported electrodes.

**Table 2:** Comparison of the proposed sensor with other previously reported electrochemical methods for SER detection.

Sensor	Analytical method	Linear Range (µM)	LOD (µM)	Reference
Fe _3_O_4_ /CSs/GCE	DPV	0.20–25.0	4.00 × 10^−3^	^2^
MnO _2_ -GR/GCE	SWV	0.10–800	0.01	^81^
Poly(BCP)MPGE	CV	10.0 − 70.0	0.49	^34^
P(L-cys)/GCE	SWV	8.00–0.30	1.99 × 10^−3^	^51^
ZNPs/MCPE	DPV	10.0 − 50.0	558 × 10^−3^	^6^
SPCE/TiO _2_ NP/AuNP/ PNB _DES_	DPV	0.30–20.0	2.40 × 10^−3^	^1^
ZrO _2_ –CuO–CeO _2_ /Gr/CPE	DPV	8.00 × 10^−3^– 205	3.49 × 10^−3^	^62^
FeC-AuNPs-MWCNT/SPCE	SWV	0.05–20.0	17.0 × 10^−3^	^23^
Graphene/AuNP/Casein/GCE	DPV	0.30–3.00	0.10	^82^
NiNPs-rGO/GCE	DPV	0.02–2.00	0.01	^31^
Nafion/Ni(OH) _2_ /MWCNTs/GCE	DPV	8.00 × 10^−3^ – 10.0	3.00 × 10^−3^	^83^
GCE/P-Arg/ErGO/AuNP	DPV	0.01–0.50, and1.00– 10.0	0.03	^75^
Fe _3_O_4_ –MWCNT–poly(BCG)/GCE	DPV	0.50–100	0.08	^45^
p(P _3_ CA)/PGE	AdDPSV	0.01–1.00	2.50 × 10^−3^	^84^
MnFe _2_O_4_ /GCN/GCE	DPV	0.10–522.60	3.00 × 10^−3^	^57^
rGO-Ag _2_ Se/GCE	DPV	0.10–15.0	29.6 × 10^−3^	^42^
PGE/AuNPs/P(L-Lys)-GQD	DPV	0.05–200	17.0 × 10^−3^	^33^
p-AZ/MWCNT-GR/CFMEA	DPV	0.10–100	13.4 × 10^−3^	^39^
Poly (FSBF)MPGE	CV	10.0–50.0	1.70	^35^
AA-MWCNT/OO-PEDOT/PGE	DPV	0.10–100	92.0 × 10^−3^	^36^
SPCE/ZnONR/PMB _DES_ /AuNP	DPV	0.10–25.0	1.90 × 10^−3^	^32^
GCE-PEDOT-AuNPs	CV	10.0–320	5.70	^85^
Poly (HA)/MWCNTs/BGE	DPV	3.00 × 10^−3^–1.00, and 5.00 − 1.00 × 10^3^	0.06 × 10^−2^	This work

(AA-MWCNT) Acid-Activated Multiwalled Carbon Nanotube, (AdDPSV) Adsorptive differential pulse stripping voltammetry, (BCG) (bromocresol green), (BCP) Bromocresol purple, (CFMEA) Carbon fiber microelectrode array, (CSs) Carbon spheres, (FeC) ferrocene, (FSBF) fast sulphone black F, (GQD) Graphene Quantum Dot, (GCN) graphitic carbon nitride, (MCPE) Modified Carbon Paste Electrode, (OO-PEDOT) Over-Oxidized Poly(3,4-ethylenedioxythiophene), (p-AZ) poly-alizarin, (P-Arg) poly(L-arginine), (PGE) Pencil graphite electrode, P(L-Lys) poly(L-lysine), P(L-cys) Poly (L-Cysteine), (PMB_DES_) polymethylene blue (deep eutectic solvent), (PNB_DES_) poly Nile blue (in deep eutectic solvent), p(P_3_CA) Poly(pyrrole-3-carboxylic acid), (rGO) reduced graphene oxide, (SPCE) Screen Printed Carbon Electrode, (ZNPs) zirconium oxide (zirconia) (ZrO_2_) nanoparticles, (ZnONR) Zinc oxide nanorod.

The poly(HA)/MWCNTs/BGE sensor repeatability was examined by recording five successive measurements DPV of 10 µM SER using a same electrode. The results showed a good repeatability with relative standard deviation (RSD) of 2.59%. While the reproducibility of the poly (HA)/MWCNTs/BGE was also tested through preparing three different modified sensors and comparing their response towards a 10 µM SER. The RSD was found to be 1.14% indicating the excellent reproducibility of the electrode.

As shown in Fig. 5E, the storage stability of the proposed sensor was tested at 4^◦^C in a refrigerator at different time intervals over10, 20 and 30 days and the sensor was found to retain 96.19% of its initial response current towards a 10 µM SER up to 20 days and 95.23% after 30 days, illustrating the good shelf stability of the prepared sensor.

### Analytical applications

SER in human blood serum samples, was determined using poly (HA)/MWCNTs/BGE sensor. Serum samples were diluted 100 times with 0.1 M PBS pH 7 at four different spiked concentrations of SER added to the diluted human blood serum sample using the standard additions method^79,80^. The results given in Table 3, show acceptable values for the recoveries and relative standard deviations (*n* = 3) in the range of 98.31 − 105.47% and 3.02–4.77%, respectively indicating that poly (HA)/MWCNTs/BGE sensor has good sensitivity and highly capability of detecting SER in biological samples.

**Table 3:** Determination of SER in human serum sample at poly (HA)/MWCNTs modified BGE.

Sample	Spiked (µM)	Found (µM)	Recovery (%) ± SD	RSD (%)
Serum sample	0.10	0.11	105.47 ± 0.005	4.77
	0.30	0.31	102.91 ± 0.011	3.78
	1.00	1.00	100.70 ± 0.040	4.02
	5.00	4.91	98.31 ± 0.148	3.02

SD: Standard deviation (n = 3); RSD: Relative standard deviation (n = 3).

SD: Standard deviation (*n* = 3); RSD: Relative standard deviation (*n* = 3).

Tryptophan (TRP), also known as 2-amino-3-(1 H-indole-3-yl) propanoic acid, is one of the essential amino acids for human health. It plays a crucial role in various biological processes, including metabolism, protein synthesis, and neurotransmitter production^86^. Since the human body cannot synthesize TRP on its own, it must be obtained through food or complementary sources^37,62^. TRP is also involved in the synthesis of niacin, a precursor to key neurochemicals like melatonin and serotonin^87^. Maintaining optimal levels of L-tryptophan is crucial, as its deficiency can lead to metabolic disorders and neurological diseases like anxiety, depression, and insomnia. On the other hand, excessive intake of TRP can result in severe health issues such as headaches, nausea, lethargy, liver problems, and even schizophrenia^86^.

In light of this, the presented poly (HA)/MWCNTs/BGE sensor was also utilized for the detection of serotonin in the presence of tryptophan. Their electrochemical behaviours were investigated using the DPV in a solution containing a mixture of SER and TRP at different concentrations ranging from 5.00 to 1.00 × 10^3^ µM (0.88–176.22 µg/ml) prepared in PBS pH 7. The DPVs have shown good separation of the peaks of SER and TRP on the surface of the modified sensor as presented in Fig. 5D. The peak current for TRP was found to increase linearly with its concentration with a regression equation represented as: І_TRP_ = −59.67 + 86.89 log[TRP] and (R^2^ = 0.992), as shown in inset of Fig. 5D.

Accordingly, it can be depicted that the fabricated sensor can be successfully applied for the detection of SER in the presence of TRP with high sensitivity, selectivity and repeatability. This can be attributed to the poly (HA)/MWCNTs/BGE sensor’s efficiency, cost-effectiveness, besides its simplicity and ease of fabrication, as well as, its excellent reproducibility and a high electrocatalytic activity.

### Greenness evaluation

The concept of Green Analytical Chemistry (GAC) emphasizes the enhancement of analytical methods to be more environmentally friendly and safer for human health. This involves reducing the quantities and toxicity of reagents, generating minimal waste, minimizing pollution, safer solvents and auxiliaries and decreasing the usage of hazardous chemicals. Current trends in green analytical method development include the development of solvent-free or solvent-reduced extraction techniques and the miniaturization of sample preparation instruments^88,89^. Recently, researchers have been dedicated to create eco-friendly analytical methods based on green chemistry principles to prevent pollution. The developed methods must adhere to green chemistry principles to ensure ecological compatibility^90^. Five green metric tools were chosen for assessing the greenness and eco-friendliness of the proposed method, namely: National Environmental Methods Index (NEMI), Raynie and Driver, Green Analytical Procedure Index (GAPI), and Analytical Greenness (AGREE).

NEMI, is the oldest qualitative tool for evaluating the greenness of analytical procedures and is represented as a pictogram with a four-quadrant circle. Each quadrant of the circle represents a different criterion: PBT (Persistent, Bioaccumulative and Toxic), corrosive, hazardous, and waste generation. If a set of criteria satisfies four principles such as having chemicals not classified as PBT, having anon-corrosive working pH, being non-hazardous, and waste production less than 50 g, the corresponding quadrants are coloured green^90,91^.

**Fig. 6:**
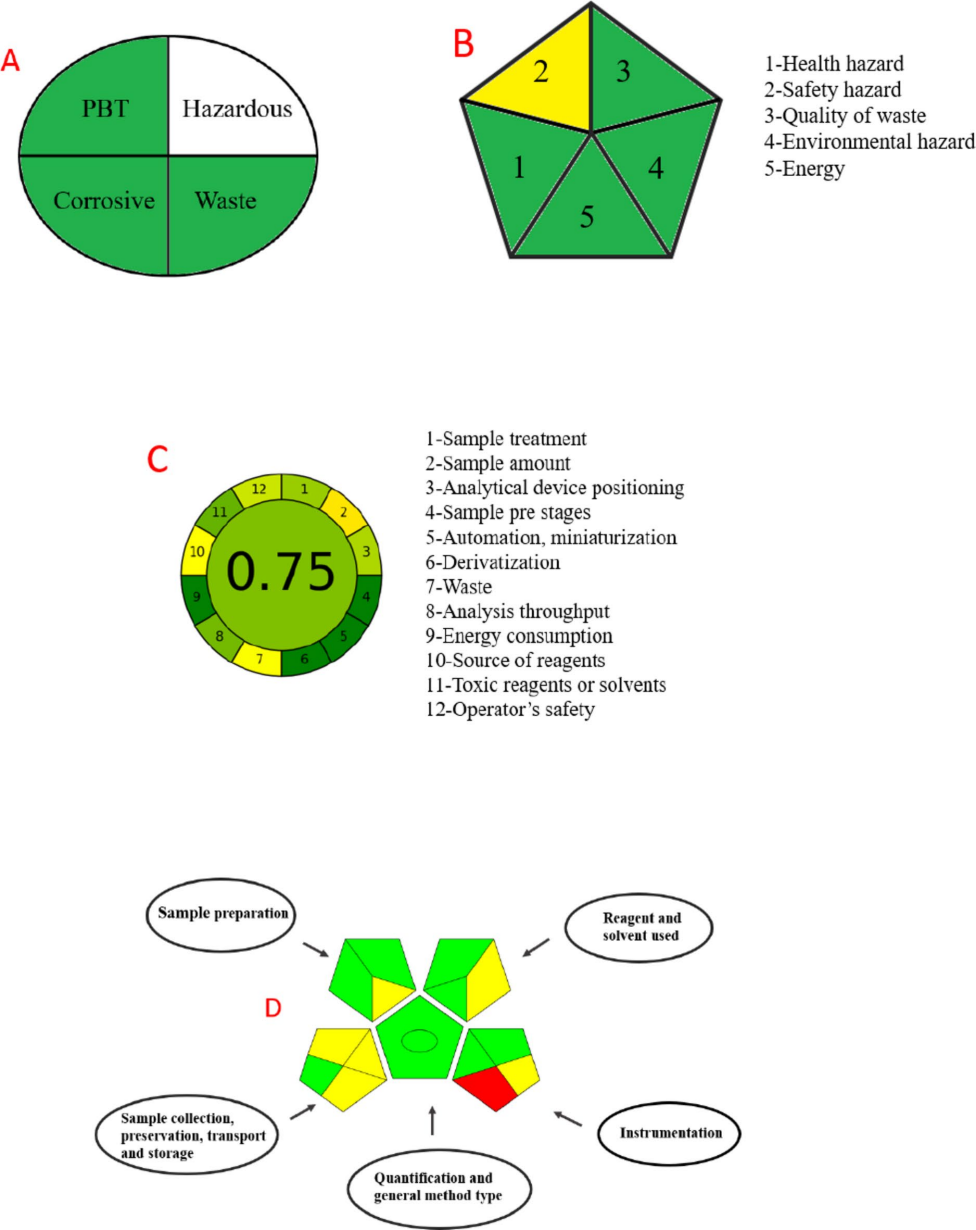
Green metric tools for assessing greenness of the proposed method (**A**) NEMI, (**B**) Modified NEMI, (**C**) AGREE, and (**D**) GAPI.

In the currently proposed method, the chemicals used were not classified as PBT, the methodology’s pH was 7.0, and the amount of waste produced was less than 50 g, resulting in three green quadrants and one white quadrant due to the use of hazardous DMF in suspending MWCNT, yet still with a very small amount. Since the procedures met NEMI requirements, the method proposed can be considered as environmental friendly and claimed to be green, as shown in Fig. 6A.

The modified NEMI or Raynie and Driver is a pentagonal pictogram divided into five portions, each representing a distinct criterion: health, safety, quality of waste, environment, and energy usage. The pictogram uses green, yellow, and red colours to indicate the environmental friendliness of the processes from low to high impact^90^, respectively. The greenness of the developed approach is highlighted by the pentagon-shaped pictogram as which contain four green triangles and one yellow triangle^88^ as represented in Fig. 6B.

Analytical Greenness (AGREE), is a modern metric tool that assesses the greenness of analytical procedures by incorporating the 12 principles of green chemistry^92^. It provides both qualitative and quantitative evaluations of a method’s greenness. AGREE is depicted as a circular pictogram divided into 12 segments, each representing a different green principle with colours ranging from green to red based on the method conditions. It is comprehensive, flexible (allowing for weight assignment), easy to use (with freely available software), and straightforward to interpret (with a coloured pictogram output). The input criteria reflect the 12 green principles, and different weights can be assigned for flexibility. The output is a clock-like coloured graph displaying the final score and colour representation in the middle as illustrated in Fig. 6C. The proposed method received a score of 0.75 without any red sections, indicating a green performance for the proposed sensor.

The Green Analytical Procedure Index (GAPI), is represented by a pentagonal pictograms with red, yellow, and green colours where green represents high level of greenness and red indicates the lowest and consisting of five pentagrams that cover sample preparation and analysis, sample collection and preservation, method type, solvents/reagents used, and instrumentation to classify a tested method as green or non-green^90,91^.

The proposed method was characterized by a short analysis time, ease of device transport, minimal waste generation, minimal energy consumption, and straightforward procedures without additional steps or specific storage conditions for samples. Consequently, the proposed method was confirmed as environmentally friendly, as shown in Fig. 6D which displayed eight green sections, six yellow section, and one red section.

A further quantitative approach is the Analytical Eco-Scale, utilized for evaluating the greenness, environmental sustainability and eco-friendliness of analytical methodologies. This method is based on penalty point score, where the penalty points are subtracted from a total scale of 100. The penalty points are determined based on the solvents or reagents used, instrument energy consumption, occupational hazards, and waste production. Additionally, the quantity of hazard pictograms provided by the Globally Harmonized System of Classification and Labelling of Chemicals (GHS) and safety data sheets for each chemical or solvent are considered. According to the Analytical Eco-Scale, a score above 75 indicates an excellent green approach, while a score above 50 indicates good green approach and a score below 50 indicates a non-eco-friendly method^90,93,94^. Consequently, the proposed method is classified as an excellent green method, achieving a score of 84, as detailed in Table 4.

**Table 4:** Analytical Eco-scale tool for the suggested method for assessing greenness.

Hazard		Penalty point
Reagents	Deionized water	0
	Phosphate buffer	4
	Dimethylformamide (DMF )	6
	Isopropanol	4
	NaOH	2
Instruments	Energy	< 0.1 kWh per sample
	Occupational Hazard (Analytical process hermitization)	0
	Wastes	1–10 mL (no treatment)
Total penalty points	16	
Analytical Eco-Scale total score^a^	84	

The overall score of the Analytical eco-scale = 100 – total penalty points., if the score greater than 75, it indicates excellent green analysis, if the score greater than 50, it indicates acceptable green analysis, and if the score is less than 50, it indicates non green analysis.

## Conclusions

For the first time, an innovative electrochemical sensor based on conducting polymeric layer of poly (HA) over MWCNTs on BGE is presented. The poly (HA)/MWCNTs/BGE sensor demonstrated its highest electrochemical performance under optimized condition including 7µL of MWCNTs, 10 electropolymerization cycles of hippuric acid, with an accumulation time of 120s and 0.01 V as the optimum potential step. SER was carried out in 0.1 M PBS at pH 7, which exhibited the maximum peak current. This novel approach facilitates the detection of SER, resulting in enhanced response and improved sensitivity. The proposed sensor showed two linearity ranges 3.00 × 10^−3^ to 1.00 µM (5.29 × 10^−4^ – 0.18 µg/ml) and 5.00 to 1.00 × 10^3^ µM (0.88− 176.22 µg/ml) for SER detection with a LOD of 0.06 × 10^−2^ µM (1.06 × 10^−4^ µg/ml) and a LOQ of 2.00 × 10^−3^ µM (3.52 × 10^−4^ µg/ml). The constructed sensor demonstrated good reproducibility, repeatability and high selectivity towards SER in the presence of various biological interferents and exhibited satisfactory recoveries ranging from 98.31 to 105.47% and RSD 3.02% – 4.77 with a stability of 96.19% after 10, 20 days and 95.23% after 30 days of its initial response current and confirming its acceptable shelf life stability.

It is noteworthy to mention that the application of Green Analytical Chemistry (GAC) evaluation tools emphasized that the proposed sensor is environmentally friendly and comply with the required tested parameters for a green method with an Analytical Eco-Scale of 84 and thus can be classified as an excellent green approach.

## Data Availability

Data and materials will be available upon request from the corresponding author Rasha M. El Nashar.
